# Morning light therapy for juvenile depression and severe mood dysregulation: study protocol for a randomized controlled trial

**DOI:** 10.1186/1745-6215-14-178

**Published:** 2013-06-17

**Authors:** Sarah Bogen, Tanja Legenbauer, Thorsten Bogen, Stephanie Gest, Thomas Jensch, Silvia Schneider, Martin Holtmann

**Affiliations:** 1 Hospital for Child and Adolescent Psychiatry, LWL University Hospital of the Ruhr-University Bochum, Hamm, Germany; 2 Department of Psychology, Ruhr-University Bochum, Bochum, Germany

**Keywords:** Bright-light therapy, Depression, Adolescents, Sleep disturbances, Severe mood dysregulation

## Abstract

**Background:**

The prevalence of depression in young people is increasing. The predominant co-morbidities of juvenile depression include sleep disturbances and persistent problems with the sleep-wake rhythm, which have shown to influence treatment outcomes negatively. Severe mood dysregulation is another condition that includes depressive symptoms and problems with the sleep-wake rhythm. Patients with severe mood dysregulation show symptoms of depression, reduced need for sleep, and disturbances in circadian functioning which negatively affect both disorder-specific symptoms and daytime functioning. One approach to treating both depression and problems with the sleep-wake rhythm is the use of light therapy. Light therapy is now a standard therapy for ameliorating symptoms of seasonal affective disorder and depression in adults, but has not yet been investigated in children and adolescents. In this trial, the effects of 2 weeks of morning bright-light therapy on juvenile depression and severe mood dysregulation will be evaluated.

**Methods/design:**

A total of 60 patients with depression, aged between 12 and 18 years, in some cases presenting additional symptoms of affective dysregulation, will be included in this trial. Morning bright-light therapy will be implemented for 2 weeks (10 sessions of 45 minutes each), either with ‘active’ light (10,000 lux) or ‘inactive’ light (100 lux). A comprehensive test battery will be conducted before and after treatment and at follow-up 3 weeks later, to assess depression severity, sleep, and attention parameters. Melatonin levels will be measured by assessing the Dim Light Melatonin Onset.

**Discussion:**

In this pilot study, the use of morning bright-light therapy for juvenile depression and severe mood dysregulation shall be evaluated and discussed.

**Trials registration:**

Current Controlled Trials ISRCTN89305231

## Background

Major depressive disorder (MDD) is one of the most prominent health problems affecting mood as well as mental and physical conditions. About 1 out of 10 adults experiences affective disturbances and a loss of interest, the core symptoms of depression [1]. Behavioral and cognitive difficulties, including memory or attention problems, changes in eating behavior, and persistent sleep disturbances, have been reported [2-4]. Effective treatment strategies for MDD in adults include antidepressant medication, psychotherapy, or a combination of both [5]. In adolescents, prevalence rates for MDD, also called juvenile depression in this group, are about 4 to 8%, and the rate seems to be increasing [6]. This relatively high rate is of particular interest for two reasons.

First, the presentation of MDD in young people might be different from that in adults. However, there are many symptoms common to both groups, including irritability, low frustration tolerance, violent temper, and externalizing and histrionic behavior [7]. These symptoms can lead to serious negative psychosocial consequences, including impaired academic and occupational functioning, high-risk sexual activity, and social difficulties [8]. Moreover, the predominant symptoms of juvenile depression implicate sleep disturbances and persistent problems with the sleep-wake rhythm [9,10], which are present in about 75% of adolescents with depression [11].

Second, treating juvenile depression is difficult. The available treatments have substantial shortcomings, and remission rates of moderate to severe depression are low [12,13]. Although cognitive behavioral therapy (CBT) is the treatment of choice for mild depression, CBT alone might not be sufficient in more severe cases. Several randomized controlled trials (RCTs) have indicated no or only small differences between pharmacotherapy with a selective serotonin reuptake inhibitor (SSRI), psychotherapy, and placebo (for an overview, see the American Academy of Child and Adolescent Psychiatry website [14]). Combination trials of both SSRIs and psychotherapy, a treatment that is commonly implemented in clinical practice, have not shown superiority to treatment with either therapy alone, and remission rates have been reported to be less than 40% [14,15]. Another important point is that treatment research has primarily focused on acute treatment, whereas early-onset depression is often chronic and recurrent. One influencing factor might be the presence of co-occurring sleep disturbances, which often remain as a residual symptom even after successful acute treatment [13]. These problems with the sleep-wake rhythm have been suggested to be a robust risk factor for the development of both the first depressive episode and recurrent episodes [1]. Sleep disturbances and insomnia presenting in juvenile depression are associated with higher depression severity, greater fatigue, and higher rates of suicidal behavior [16]. Emslie *et al*. noted in two large, double-blind RCTs [17] that sleep disturbances may negatively affect treatment response, as adolescents receiving treatment with the SSRI fluoxetine were less likely to respond to the treatment if they also had sleep disturbances. Based on these results, it is essential that sleep disturbances are adequately assessed and co-treated consistently along with the depression. However, there is as yet no evidence-based treatment for insomnia in adolescents with depression [17].

One possible non-pharmacological treatment approach for ameliorating depressive symptoms and co-occurring sleep disturbances is morning bright-light therapy, which has been used successfully for adults [18,19]. Light therapy has primarily been studied in patients with seasonal affective disorder (SAD) and has been shown to have good efficacy in ameliorating depressive symptoms in this subgroup. In adults with SAD, Lam *et al*. [20] reported that light therapy was as efficient as antidepressant treatment with fluoxetine, but had a faster onset of action and fewer side effects. The effects of light therapy are apparent after about 1 week of treatment [21], and remission rates of up to 80% have been reported for SAD [22]. In addition, Even and colleagues [19] carried out a systematic review based on 15 studies, and reported efficacy of light therapy as an adjuvant treatment to antidepressants in non-seasonal depression as well. However, they concluded that the evidence for the effects of light therapy alone (without antidepressant) was still inconsistent [19].

Light therapy has also been shown to be effective for other symptoms besides depression. For example, it may induce stabilization of the circadian rhythm (the biological rhythm controlling the sleep-wake cycle) and thereby, improve difficulties with sleep onset and difficulties sleeping through the night [23]. This is of particular interest because an intimate relationship between sleep and emotion regulation has been reported [24], with the consequences of disturbed sleep including symptoms such as heightened impulsivity and aggressive behavior. Preliminary evidence indicates that light therapy has a positive influence on behavior, irritability and attention parameters [25-27]. Furthermore, it has been suggested that, independent of specific diagnoses, the severity of psychiatric symptoms increases and long-term outcomes worsen when circadian disturbances are present [28], reinforcing the crucial relationship between sleep and regulation of emotions. Therefore, it is reasonable to assume that light therapy might be a useful method of stabilizing circadian functioning and thereby inducing more general improvements on emotional regulation. Despite these positive results in adults, there have only been very few studies investigating light therapy for adolescents. One RCT of children and adolescents with SAD showed that 1 week of light therapy significantly decreased parent-rated depressive symptoms [29]. A more recent 1-week trial of light therapy as an adjunctive treatment for young people with mild depression showed significant improvements in depression scores on the Beck Depression Inventory (BDI) from baseline to the end of therapy in the active treatment group [30]; however, participants received concomitant CBT and pharmacotherapy during the trial, which may have led to additional positive effects.

One phenotype that has a high prevalence rate in clinical populations and that elicits considerable problems with emotional regulation, depression, and circadian disturbances is severe mood dysregulation (SMD). Children and adolescents with SMD show severe affective and behavioral dysregulation, including irritable mood, hyperarousal, and increased reactivity to negative emotional stimuli [31,32]. Characteristics of SMD include not only depressive symptoms that might develop into MDD later on [33], but also circadian dysfunctions such as reduced need for sleep, disturbances in sleep continuity at sleep onset and through the night, lower sleep efficiency, reduced rapid eye-movement (REM) sleep and impaired daytime behavior, which have consistently been reported [34-36]. The initial treatment approaches, similar to those for pediatric bipolar disorder and attention deficit hyperactivity disorder (ADHD), include mood stabilizers and stimulants, and have shown some positive effects [37,38]. A novel psychosocial treatment with CBT indicated improvements in depressive symptoms, mood lability, and global functioning [39]. Although these initial treatment approaches were reported to have some positive effects on SMD symptoms, children and adolescents with SMD and additional ADHD were more likely to remain significantly impaired than those with only ADHD after a 3-week combination trial with stimulants and behavior modification therapy [37]. Therefore, a wider range of treatment approaches for SMD is needed, as those that have been evaluated to date have shown only limited improvements. It is possible that, as in juvenile depression, circadian disturbances might be an influencing factor on treatment outcome in SMD. Considering that preliminary results of light application besides SAD and MDD have shown positive influences on affective and behavioral regulation and on circadian functioning, light therapy might constitute a reasonable co-treatment for SMD symptoms as well [40].

The proposed study is an RCT of bright-light therapy in juvenile depression. We plan to enroll 60 adolescents with depression. We hypothesize that 2 weeks of morning bright-light therapy will improve depressive symptoms and additional sleep disturbances in these adolescents. On an exploratory basis, the study will additionally evaluate the outcomes of morning light therapy on SMD symptoms.

## Methods/design

### Participants and recruitment

Participants of this study will be recruited from the inpatient unit of the LWL (University Hospital Hamm, Ruhr University Bochum, Germany), a tertiary-care hospital for child and adolescent psychiatry. The hospital provides child psychiatric care for a population of 1.6 million inhabitants, covering both urban and rural areas, and is the sole provider of inpatient care serving the study area.

All patients between 12 and 18 years who are referred to the hospital will be screened consecutively for participation. Patients with moderate or severe depression (according to the International Classification of Diseases, version 10; (ICD-10), based on parent and child interviews and assessed using the second edition of the BDI (BDI-II [41]), will be included in the trial. Diagnoses will be confirmed using the Diagnostic Checklist for Depressive Disorders [42] conducted by a clinical psychologist or child psychiatrist. Presence of co-morbid disorders will be allowed, excluding bipolar 1 disorder and schizophrenia. The specific eligibility criteria of the study are listed in Table 1.

**Table 1:** Eligibility criteria

Inclusion criteria	Moderate to severe depression (ICD-10/BDI-II)
	Being 12 to 18 years of age
	Ability to understand character and individual consequences of the trial
	Written informed consent of the person with primary custody must be available before enrolment in the trial
Exclusion criteria	Acute suicidal tendencies
	Treatment with antidepressants
	Treatment with beta-blockers
	Treatment with high-potency neuroleptic drugs
	Diagnosis of bipolar 1 disorder or schizophrenia
	Diseases of the eye with involvement of the retina
	Pregnancy or lactation

BDI-II, Beck Depression Inventory, second edition; ICD-10, International Classification of Diseases, version 10.

#### Ethics and written consent

The study has been approved by the medical ethics committee of the Ruhr-University Bochum, Germany (registration number 3996–11), and will be conducted in accordance with the Helsinki Declaration. Before entering the study patients will be informed about the study objectives, study design, and potential risks by one of the main investigators and will also receive this information in writing. Patients will be informed about their right to withdraw from the study at any time. Written consent will be obtained from the persons in charge with primary custody. After informed consent is obtained, the therapist in charge will be informed by mail about the patient entering the study.

#### Randomization and blinding

After screening, patients will be randomized and allocated to either the active light intervention (experimental group; 10,000 lux) or to the inactive light intervention (control group; 100 lux). Randomization will be carried out by randomly drawing group allocations by hand from a randomizer box in which the number of possible group allocations is equal. For practical reasons, the primary investigator of the study who is performing the randomization and delivering the treatments (but is not involved in outcome ratings) will be aware of the participants´ allocation, but all therapists on the patients´ units will be blinded to the allocation groups.

### Interventions

Interventions will be conducted following the protocols described by Wirz-Justice *et al*. [43]). Based on empirical evidence indicating good results for light therapy with a duration of 7 to 14 days for non-seasonal depression [18,19], two parallel interventions will be created, each lasting 2 weeks, and will be conducted in the mornings, in addition to usual treatment in the respective treatment units.

#### Experimental group

Patients randomly allocated to the experimental group will receive 2 weeks of active morning light therapy with 10,000 lux at eye level in gaze direction (LD 110^®^; DAVITA^®^ Medizinische Produkte GmbH & Co. KG, Kleve, Germany), with each session lasting for 45 minutes. The therapy will start about 7.5 hours after the estimated individual dim light melatonin onset (DLMO), estimated by the Morningness-Eveningness-Questionnaire (MEQ) [44]), as it has been shown that by administering light at that time point, the efficacy of light therapy is increased [22].

#### Control group

A valid placebo group will be created by randomly assigning half of the patients to the control group to receive 2 weeks of inactive light therapy not exceeding 100 lux at eye level in gaze direction (Luxor LED light alarm clock, lowest level; DAVITA^®^ Medizinische Produkte GmbH & Co. KG, Kleve, Germany), with each session lasting for 45 minutes. Each session will commence about 7.5 h after the estimated DLMO to parallel the experimental group.

Finding an appropriate control group for light therapy to minimize placebo effects is difficult, because these effects are expected to be relatively high [19,45,46]. To design conditions that the patients will not consider a control group, we have chosen professional light-therapy devices for both groups, adjusting the lux levels respectively. To control for possible expectation bias, both groups will be instructed that the aim of the study is to compare two different types of light therapy. Moreover, to minimize bias, the therapists on the respective units will be informed that the light therapy for both groups is active.

To ensure that an appropriate and constant number of lux is administered in each group, intensities will be quantified using a lux meter (PCE-172; PCE Instruments, Meschede, Germany). The light intensity that will be used in this study lies within the range of normal daylight exposure. Its long-term ocular safety has been shown in patients with SAD who received bright-light therapy at an illuminance level of 10,000 lux [47].

**Figure 1:**
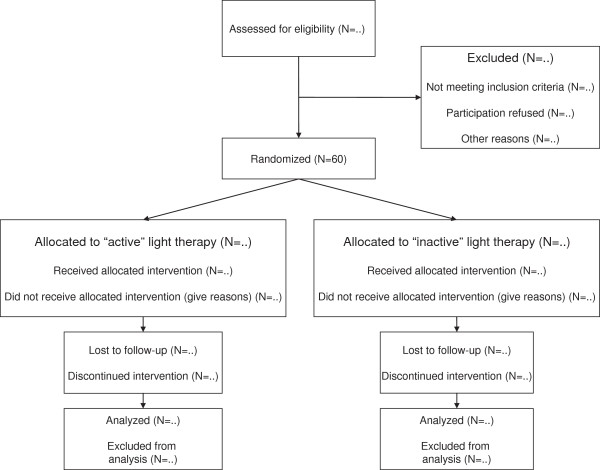
Study flow chart.

During the interventions, patients will be allowed to have breakfast or read magazines and books with neutral content (for example, magazines containing plain information without emotionally arousing material). To ensure that every participant has received the same amount of light therapy at the end of the treatment period, treatment can be prolonged for up to 1 day if a patient misses a light-therapy session for some reason (for example, somatic illness). If a patient misses more than two sessions, he/she will be excluded from the study. In total, about three patients can be treated in parallel within each group (see Figure 1 for a flow chart of the study).

### Primary and secondary endpoints

The primary endpoint of the study is the change in depression symptoms (BDI-II total score) during the trial. Secondary endpoints include changes in general psychopathology, sleep, circadian functioning (including melatonin assessment), affective dysregulation, behavior, and attention parameters. All questionnaires will be presented in German, and will be completed within the hospital.

#### Assessments

##### BDI-II

BDI-II is a measure of the severity of depressive symptoms and has been used widely in treatment studies for juvenile depression [41]. It can be used to directly compare effect sizes between different kinds of interventions. The German version has shown good psychometric properties, with good test-retest-reliability (*r* ≥ 0.75) in a non-clinical population, and good internal consistency (α coefficient = 0.92) for adolescent inpatients with depression [48,49].

##### Clinical global impression scale

Assessment of general psychopathology will be performed using the Clinical Global Impression Scale (CGI) for estimating symptom severity (CGI-S) and improvement (CGI-I) [50,51]. The CGI is a 7-point scale that requires a rating of illness severity at the time of assessment. Because severity estimation in the CGI is performed in relation to other patients, it is a subjective assessment tool. In the proposed study, rating will be performed by clinicians blinded to group allocation.

##### Sleep questionnaire B - revised

Sleep and sleep difficulties will be assessed by using the Sleep Questionnaire (Schlaffragebogen) B - Revised (SF B/R) described by Görtelmeyer *et al*. [52]. This questionnaire contains 31 questions, assessing the 2 weeks prior to assessment. Completion takes about 5 to 10 minutes, and 11 sleep indices can be created including: difficulties in sleep onset and in sleeping through the night, premature awakening, general sleep characteristics, total sleep duration, and sleep factors consisting of sleep quality, feeling of recovery after sleeping, mental balance before going to sleep, feeling of mental exhaustion before going to sleep, and psychosomatic symptoms during sleep phase. Internal consistency is moderate to excellent (α coefficient = 0.68 to 0.92) for clinical populations. Test-retest reliability indicates that relatively stable sleep-related behavior and experiences can be assessed (r = 0.53 to r = 0.91).

##### DLMO

Melatonin production by the pineal gland is under the influence of the suprachiasmatic nucleus of the hypothalamus (SCN), which receives information from the retina about daily patterns of light and darkness [53]. Production of melatonin is inhibited by light and induced by darkness. Hence, when dim lighting is provided, the human body starts producing melatonin about 2 hours before bedtime, and this physiological process is called DLMO. Assessment of the DLMO can be considered the gold standard for measuring melatonin levels and circadian rhythms. In the present study, at the assessments T1, T2, and T5, four sequential melatonin saliva samples will be collected at hourly intervals, respectively. Collection starts from 4 hours before predicted bedtime (19.00 to 22.00 hours) under conditions of dim light. Light therapy has been shown to be most effective when it is applied about 7.5 to 9.5 hours after the individual DLMO [22].

Saliva will be sampled using sterile collection devices (Salivettes; Sarstedt AG & Co, Nümbrecht, Germany). On the day of sampling, patients will be instructed to avoid caffeinated beverages, orange juice, eggs, chocolate, and bananas, because all these have been shown to have a possible influence on melatonin level. During the evening, electronic devies such as video games, TV, and cell phones will not be allowed because the light produced by such devices may have a melatonin-suppressing effect [54]. Patients will be instructed to refrain from food or drinks for 30 minutes before sampling, and directly before sampling they will be instructed to rinse their mouth with water. Samples will be stored at −26°C until analysis, and all samples will be analyzed simultaneously. This will be performed in a specified laboratory in which melatonin concentrations will be determined, and the individual DLMO will be calculated.

##### Morningness-eveningness questionnaire

For estimating circadian preferences independent of physiological parameters, the German version of the Morningness-Eveningness Questionnaire (MEQ) will be used [44,55]. With this questionnaire, the individual chronotype can be estimated based upon 19 questions that are summed to give a total score and the individual DLMO is then estimated, as it has been shown to have a good correlation with the MEQ [56]. Using this procedure, the optimal time point for light exposure can be estimated directly (about 7.5 h after the individual DLMO) without the requirement for physiological data.

##### Child behavior checklist

The Child Behavior Checklist (CBCL) is one of the best-studied, empirically derived parent checklists for measuring general child and adolescent psychopathology [57]. It assesses the child’s behavior over the past 6 months using a total of 118 items (plus two optional questions), and is rated by the parents or primary caregivers. The questionnaire includes a total problem score, two higher level scales (externalizing problems and internalizing problems), and eight syndrome scales (withdrawn, somatic complaints, anxious/depressed, social problems, thought problems, attention problems, delinquent behavior, and aggressive behavior). The reliability, factorial validity and discriminant validity of the German adaptation of the CBCL have been confirmed by previous studies [58,59]. Recently, a specific ‘dysregulation profile’ has been identified in the CBCL, capturing severe affective dysregulation (CBCL-DP) [60]. This profile is characterized by simultaneous high values on the syndrome scales ‘anxious/depressed,’ ‘attention problems,’ and aggressive ‘behavior.’ The CBCL-DP has been consistently found to be associated with disruptive behavior disorders, suicidal behavior, and reduced need for sleep [61-64], and it will be calculated to assess the phenotype of affective dysregulation.

##### Strengths and difficulties questionnaire

In addition to the CBCL, the Strengths and Difficulties Questionnaire (SDQ) will be used in order to assess short-term changes and to evaluate parent and patient ratings. The SDQ is a brief behavioral screening questionnaire assessing 25 attributes, some positive and others negative, which can be allocated to five scales (emotional symptoms, conduct problems, hyperactivity/inattention, peer relationship problems, and pro-social behavior) [65]. These scales can be summed to calculate a total difficulties score and, like in the CBCL, a dysregulation profile can be calculated that corresponds to the CBCL-DP, with the advantage of being able to assess short-term changes [66]. In this study, patient and parent rating versions of the SDQ will be used. The sensitivity to change of the SDQ allows estimation of treatment efficacy (α = 0.73; retest stability = 0.62) [67], therebv allowing monitoring of symptom changes of affective dysregulation and behavior during the trial.

##### Tests of attentional performance

With the Tests of Attentional Performance (TAP), a large spectrum of specific attentional performances can be assessed in a computerized form [68]. The following four subtests will be administered to evaluate highly relevant attention-related parameters.

1) Covert shift of attention. This assesses the ability to shift the attentional focus, measuring reaction times for valid and invalid cues in a visual reaction time task.

2) Go/nogo. Assesses the ability to suppress a response in the presence of irrelevant stimuli.

3) Alertness. Assesses the general level of arousal (tonic arousal), its stability over a longer period (intrinsic arousal) and the magnitude of elevation of arousal, induced by a warning signal (phasic arousal).

4) Divided Attention. Assesses the ability to divide attention to auditory and visually presented target stimuli.

Internal consistency for all these subtests lies between 0.60 and 0.83 [68].

##### Seasonal pattern assessment questionnaire

The Seasonal Pattern Assessment Questionnaire (SPAQ) is a questionnaire for retrospectively assessing change in mood and vegetative functions with the seasons [69]. It is a frequently used screening instrument for assessing symptoms of SAD, with a high internal consistency [70] and has recently been used in young adults [71]. In the present study it will be administered to calculate a total seasonality score, with seasonality being the degree to which seasonal changes affect criteria such as mood, energy, sleep length, appetite, food preference, or the wish to socialize with other people [69], and thus will be used to control for seasonal effects.

##### Therapy expectancy

Therapy expectancy will be assessed by using a modification of the Credibility/Expectancy Questionnaire, which is a quick and easily administered scale for measuring treatment expectancy and rationale credibility for use in clinical outcome studies [72]. High internal consistency has been reported, lying between 0.84 and 0.85 for the whole scale. Inter-item correlations between studies ranged from 0.53 to 0.85 for the items on the expectancy factor, and between 0.62 and 0.78 for items on the credibility factor. Test-retest reliability over a 1-week period was also found to be good, at 0.82 for expectancy and 0.75 for credibility [72]. In the proposed study, the questionnaire will be used to control for placebo effects, which are expected to be relatively high for light- therapy studies.

##### Culture fair intelligence test 20 – revised

The Culture Fair Intelligence Test 20 – Revised (CFT20-R) is an economic, computerized general intelligence screening test assessing ‘general fluid ability’ [73], that is, the ability to solve formal logic tasks with varying complexity within a specific time frame. The CFT20-R uses language-free tasks and is therefore also appropriate for people with language deficiencies. It is standardized for children and adults (8.5 years of age and above) and has high test-retest coefficients (r = 0.80 to 0.83, uncorrected) and high internal consistency (α = 0.95).

##### Adverse events

At each assessment, the participants will be asked to report any adverse events (AEs). Reported AEs of light therapy side effects include jumpiness, jitteriness, headache, or nausea (for an overview, see Terman *et al*. [22]). AEs will be assessed by using open questions, asking about general AEs and their severity during the study period.

#### Time points of assessments

The assessment time points are as follows.

•T1: The initial assessment for eligible patients will be carried out after about 1 week of care in the unit. At this point, patients will have become used to being in the hospital, and will probably have a more regular day and night rhythm.

•T2: After 2 weeks of morning light-therapy intervention, there will be a comprehensive post-therapy assessment.

•T3: One week later, depression severity will be assessed to monitor changes.

•T4: Another week later, there will be another assessment of depression severity.

•T5: Three weeks after the light-therapy interventions have ended, another comprehensive assessment will be carried out.

**Figure 2:**
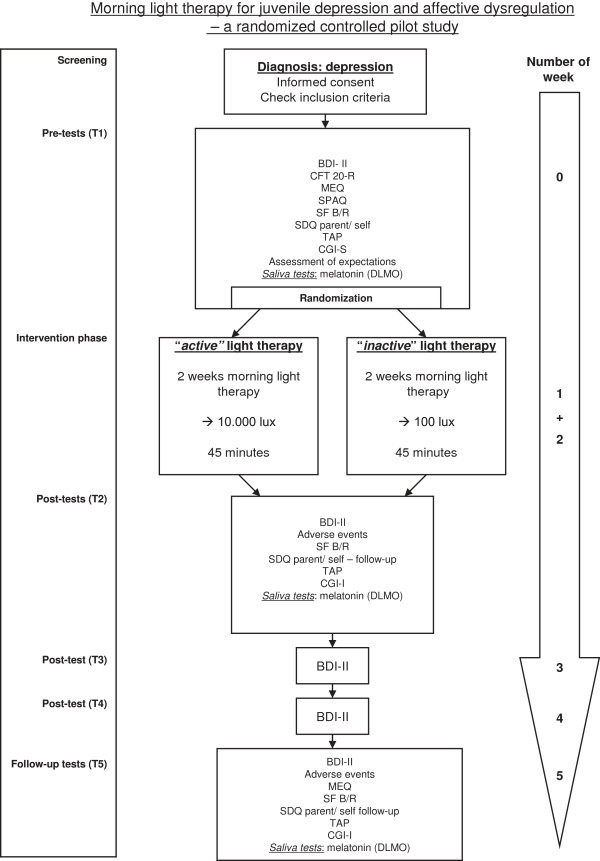
**Time schedule for the pilot study.** BDI-II, Beck Depression Inventory-II; CFT 20-R, Culture Fair Intelligence Test – Revised, CGI-S/ I, Clinical Global Impression Scale-Severity/ Improvement; DLMO, Dim Light Melatonin Onset; MEQ, Morningness-Eveningness-Questionnaire; SDQ, Strengths and Difficulties Questionnaire; SF B/R, Sleep Questionnaire B - Revised; SPAQ, Seasonal Pattern Assessment Questionnaire; TAP, Tests of Attentional Performance.

An overview of the timeline of the study is shown in Figure 2.

### Data analysis

#### Sample-size calculation

Estimates of a clinically relevant effect size were derived from a meta-analysis that evaluated effects of bright-light therapy for seasonal and non-seasonal depression in adult outpatients [74]. A similar study for adolescent inpatients is not available. Therefore, effect-size calculation will be based on evidence from adult outpatients. Although research is generally scarce, it is assumed that the active light therapy intervention for SAD will have an effect size (pre-post) of about 0.78 [75], which can be considered a conservative estimate because effect sizes might be considerably higher [74]. Golden *et al*. [74] divided the studies used in their meta-analysis into four categories, and derived effect sizes for them. In the category ‘non-seasonal depression’, an effect size of 0.53 was reported for bright-light treatment, with a 95% confidence interval of 0.18 to 0.89. Conservative calculations indicate that the expected outcome for our study will require a total sample size of 54 subjects (α = 0.05, two-sided test) to achieve a power of 80%.

#### Statistical analyses

Data will be assessed using SPSS software (version 20; SPSS Inc., Chicago, IL, USA). Correctness of the data will be assured by double-entry of the data. Data will be analyzed according to both intention-to-treat (ITT) and per-protocol (PP; efficacy population) principles. ITT will assess all randomized patients, while PP analyses will assess patients who receive 2 weeks of morning light therapy with no violation of the study protocol.

All planned comparisons will compare T2 to T5 relative to T1. The primary endpoint of the study is change in depressive symptoms (BDI-II total score) during the trial. Baseline depression level (that is, BDI-II total score at T1) will be controlled for in the two-factor repeated-measures ANCOVA. This model accounts for differences between groups at baseline. Furthermore, the study will evaluate whether the effects of morning light therapy on mood might partly be mediated by its effect on the circadian rhythm, and will be assessed by questionnaires and saliva samples. For missing values, the multiple imputations (MI) technique will be used, because a recent meta-analysis for RCTs in obesity found this to have modest superiority to fitting mixed linear models or using last observation carried forward (LOCF) methods [76]. With the MI technique, plausible values for the missing data are imputed. Each of the datasets is then analyzed separately using the desired model (in this case, the two-factor repeated-measures ANCOVA). With this parameter, estimates are made for each of the datasets, and these are then combined using standard rules for MI analyses [77,78]. These combined parameter estimates can then be used for hypothesis testing and for making inferences. In this RCT, two-factor repeated-measures ANCOVA will be used with group as between-subject factor and time as within-subject factor (T1 to T5). In cases of significant correlations with the primary endpoint, covariates will include baseline BDI-II score, gender, intelligence, seasonality, and circadian preference. The 95% confidence intervals and pre-post effect sizes will be derived for each analysis. All tests of statistical significance will be interpreted with a criterion of *P* < 0.05, two-sided.

## Discussion

We have presented a design and protocol for an RCT of light therapy for juvenile depression and severe affective and behavioral dysregulation in a naturalistic tertiary-care inpatient setting. This initial pilot study will evaluate the feasibility and acceptance of this chronobiological intervention for depressive adolescents by implementing a 2-week period of bright-light therapy. Because it will be implemented in an inpatient setting, there are several limitations, such as the short duration of bright-light therapy, interruption by weekend breaks, and lack of daily mood self-assessments that would allow a detailed picture of response to this chronobiological intervention. An additional challenge is the issue of blinding. In their systematic review, Even *et al*. [19] discussed the possibility that some patients in a control group exposed to a device that only delivers 100 lux may correctly guess that they have been allocated to the placebo group. To control for possible expectation bias, both groups in this study will be instructed that the aim of the study is to compare two different types of light therapy. In accordance with this, it is important to bear in mind that even 100 lux in the morning might have some subtle effect on the circadian system. However, the answers to the questions of whether morning light therapy will have a beneficial effect on non-seasonal depressive symptoms in young people, and whether 2 weeks of morning light will be sufficient to elicit more general positive effects on the sleep-wake rhythm and on affective dysregulation, should be answered. Moreover, we expect insight into possible changes on a physiological level, such as shifts in DLMO.

After this pilot study has been conducted and specific effects of bright-light therapy have been observed, a combination of various chronotherapeutic interventions, such as light therapy, wake therapy, and additional sleep-phase advance could be conducted to investigate effects in young people more thoroughly.

## Trial status

Ongoing trial.

## Competing interests

MH has served in an advisory or consultancy role for: Lilly, Novartis, and Bristol-Myers Squibb, and has received conference attendance support or was paid for public speaking by AstraZeneca, Janssen-Cilag, Lilly, Neuroconn, Novartis, Medice, and Shire. The present work is unrelated to the above grants and relationships. The other authors have no conflicts of interest.

## Authors’ contributions

SH conceived the research project; SH, TL, SSC, and MH designed the study; and SH, TL, SSC, and MH designed and tailored the study protocol. All authors contributed to the writing of the manuscript. All authors read and approved the final manuscript.
